# In vivo periodontal ultrasound imaging via a hockey-stick transducer and comparison to periodontal probing: a proof-of-concept study

**DOI:** 10.1007/s00784-025-06346-w

**Published:** 2025-04-26

**Authors:** Lei Fu, Jason J. Chang, Khalid Al Hezaimi, Lekshimi Sasi, Suhel Khan, Baiyan Qi, Casey Chen, Jesse V. Jokerst

**Affiliations:** 1 Aiiso Yufeng Li Family Department of Chemical and Nano Engineering, University of California, La Jolla, San Diego, CA 92093 USA; 2 Herman Ostrow School of Dentistry, University of Southern California, 925 West 34th Street, Los Angeles, CA USA; 3 Material Science and Engineering Program, University of California, La Jolla, San Diego, CA 92093 USA; 4 Radiology Department, University of California, La Jolla, San Diego, CA 92093 USA

**Keywords:** Periodontitis, Ultrasound imaging, Periodontal probing

## Abstract

**Objective:**

The objective of this study is to evaluate a compact ultrasound transducer to image anatomical biomarkers for periodontal diagnosis of teeth, including difficult-to-reach posterior teeth.

**Materials and methods:**

A 9-MHz hockey-stick transducer was used to image 53 premolars, 30 molars, and 79 incisors and canines from 13 subjects. The alveolar bone crest (ABC), cementoenamel junction (CEJ), and gingival margin (GM) were identified by ultrasound imaging. The image-based distances between these anatomic landmarks were measured for iABC (ABC to CEJ), iGR (GM to CEJ) and iGH (ABC to GM). The measurements were compared to corresponding parameters obtained from clinical examination. The measurements were also used to assess periodontal health and were compared with clinical diagnosis.

**Results:**

The average iGR measurements were − 1.12 mm (i.e., 1.12 mm above the CEJ) for gingivitis and Stage I periodontitis, and − 0.56 mm for Stage III periodontitis, demonstrating a significant increase in gingival recession in patients with severe periodontitis (Student t-test, unpaired, two-tailed, *p* < 0.0001). The iGH measurements distinguished gingivitis, Stage I periodontitis group, and the Stage III periodontitis group (unpaired, two-tailed t-test, *p* < 0.05 for PPD, and *p* = 0.05 for iGH).

**Conclusion:**

Non-invasive periodontal ultrasound imaging can be used to stratify subjects with differing periodontal disease severity. The clinical parameters obtained from ultrasound imaging with the hockey-stick transducer are reproducible.

**Clinical relevance:**

The compact ultrasound transducer can be used as a screening tool for patients affected by periodontitis for clinical examination and treatment.

**Supplementary Information:**

The online version contains supplementary material available at 10.1007/s00784-025-06346-w.

## Introduction

Periodontitis is a chronic inflammatory disease attributed to microbial dysbiosis and host response that destroy the tooth-supporting structures [1, 2]. It is one of the most common bacterial infectious diseases in humans and a global health challenge [3, 4]. Conventional approaches to periodontal diagnosis include radiography, visual inspection, and clinical probing [5]. Radiography provides good contrast on hard structures but insufficient contrast on soft tissue. Visual inspection and bleeding on probing are commonly used to assess tissue inflammation but can be subjective. Clinical probing provides quantitative assessments of gingival recession, periodontal probing depth (PPD), and clinical attachment level (CAL); these clinical parameters are critical for diagnosing and classifying periodontitis. However, clinical probing is time-consuming and invasive, and the results can be affected by the probing force, the insertion point, and the probing angulation [6]. Clinical probing also has large intra-examiner and inter-examiner variations. The standard deviation of the intra-examiner probing measurement of CAL ranged from 0.78 to 0.95 mm and even higher for inter-examiner measurement [7]. These variations preclude the use of clinical probing to detect small and incremental losses of periodontium. For example, the mean clinical attachment loss has been reported to be 0.57–0.1 mm per year [8].The loss of periodontium occurs in complex patterns that show significant variations among subjects and individual teeth [9, 10]. Therefore, monitoring periodontal disease progression remains a challenge for clinicians. Indeed, developing a non-invasive and highly accurate tool to monitor periodontal disease status will significantly advance clinical periodontology.

In recent years, ultrasound imaging has been increasingly studied for assessing peri-implant and periodontal health [11–17]. Ultrasound imaging reconstructs the structural information of tissue by receiving echoes from the tissue interface with different sonic impedances. It is non-invasive and provides good contrast at soft and hard tissue interfaces. A single ultrasound image reveals structures and anatomic landmarks including alveolar bone crest (ABC), CEJ, and gingival margin (GM). These landmarks can be used to derive clinical parameters to evaluate periodontal health and disease. For example, the CEJ is a reference point for measuring gingival recession and CAL. Ultrasound imaging can locate CEJ accurately with image resolution down to 50 μm [18], thus making it particularly suitable to assess gingival recession when the CEJ is not exposed. Also, the anatomic landmarks and features of the periodontium can be resolved in a three-dimensional (3D) view upon a single 1D scan [19, 20]. However, the measurement of PPD requires the application of contrast agents to the gingival sulcus in conjunction with ultrasound imaging [21, 22].

Moore et al. demonstrated that ultrasound imaging is a valid tool for periodontal diagnosis [18]. The study used a 40-MHz transducer and scanned 66 incisors and canines from healthy subjects (*n* = 10 people) and Stage II-IV (*n* = 6 people) periodontal patients. The ultrasound imaging with clinical probing was compared and confirmed that the image-derived clinical parameters were sufficient to distinguish periodontal health and disease. However, only incisors and premolars were studied due to the relatively large size of the transducer. Here, as a follow-up to the previous work, a commercially available 9-MHz “hockey-stick” transducer was used and 162 anterior and posterior teeth in 13 human subjects were imaged.

## Materials and methods

### Hardware

A commercially available hockey-stick transducer (ATL CL15-7, Philips) was used to image the teeth. It has a central frequency of 9 MHz and bandwidth from 7 to 15 MHz. A research-based ultrasound data acquisition system (Vantage; Verasonics, Inc., Kirkland, Washington, United States) was used to collect and process the ultrasound signal. Before imaging, we wrapped the transducer and a 4-mm-thick gel pad in Tegaderm film (3 M) [23].

### Human subjects

The University of Southern California (USC) and the University of California, San Diego (UCSD) Institutional Review Boards approved the clinical study protocol. It was in accordance with the ethical guidelines for human subjects research established by the Helsinki Declaration of 1975. All subjects provided written informed consent before imaging. The start date and end date of this study are 10/7/2022 and 1/31/2023.

Adults were recruited from patients seeking dental treatment at USC School of Dentistry. As part of the standard care, all subjects received a full examination, diagnosis and treatment plan, including a review of dental and medical history, FMX, periodontal charting. The periodontal diagnosis followed the guidelines in the 2017 Classification of Periodontal and Peri-Implant Diseases and Conditions [24].

Subjects must be healthy, non-pregnant adults weighing at least 110 pounds, with one quadrant with at least 4 teeth, including at least one incisor and one molar. The exclusion criteria were (i) subjects known to have a blood-borne pathogen infection, (ii) pregnant or lactating women, (iii) subjects with any acute oral infection (iv) patients taking anticoagulants, (v) subjects who require antibiotic prophylaxis for infective endocarditis prophylaxis, (vi) immunocompromised subjects, (vii) subjects weigh less than 110 pounds, (viii) subjects with anemia, blood cancer, or any other bleeding/clotting disorder.

### Clinical probing and diagnosis

Thirteen adult subjects (> 18 years old) were recruited for this study. A periodontist (C.C.) confirmed the periodontal diagnosis and examined the quadrants used for imaging to determine PPD, CAL, and gingival recession with a #12 Marquis probe (Henry Schein, Melville, NY) at six sites per tooth (mesio-labial, mid-labial, disto-labial, mesio-lingual, mid-lingual, and disto-lingual sites). The mid-labial measurements were used to compare with ultrasound imaging. Gingival recession was defined as the distance between CEJ and GM. The result can be a positive value (clinical recession) or a negative value (gingival tissue is coronal to the CEJ). Only the mid-labial measurements were used to compare with ultrasound imaging. Seven subjects were diagnosed with Stage III periodontitis, two with Stage I periodontitis, and four with gingivitis. All the subjects provided their written consent before imaging. A total of 162 teeth were imaged including 53 premolars, 30 molars, and 79 incisors and canines.

### Periodontal ultrasonography

Mid-labial measurements of clinical probing were used to compare with ultrasound imaging. The lingual side is not accessible due to the physical design of the hockey-stick transducer. The mesial and distal sites are not imageable by ultrasound, as the backscattered ultrasound is deflected away from the imaging plane. The images were analyzed in ImageJ without additional processing or modifications that could alter their integrity. The ultrasound images were analyzed independently by an ultrasound researcher (L.F.) and a dental student (J.C.) who also has experience in ultrasound imaging. Each examiner identified the ABC, CEJ, and GM from an ultrasound image as described previously [11, 18]. The distances between ABC, CEJ, and GM were then measured using digital image processing (ImageJ). The distances were measured when the two anatomical landmarks were both identifiable. For example, if the CEJ could not be identified, then the distance between the ABC and CEJ as well as GM and CEJ would not be measured. The image range is 22.6 mm (770 pixels, width) × 11.7 mm (400 pixels, depth). The images were loaded into ImageJ. The distances were projected onto the cementum surface and measured by using a line segment tool. The dimensions in mm and pixels of the images are preloaded into the software. A straight line was drawn between different landmarks and the distance was measured via the Measure function in the Analyze tab. The distance between ABC and CEJ is defined as imaging-based alveolar bone loss (iABL). The distance between GM and CEJ is imaging-based gingival recession (iGR). The distance between ABC and GM is imaging-based gingival height (iGH). All image quality metrics, image measurements, and clinical measurements are included in Supplementary Appendix Table.

### Statistical analysis

All the statistical analysis was performed with GraphPad Prism 9 (San Diego, CA). Student’s t-test (paired and unpaired) was used to determine differences between two sets of measurements with 95% significance (α = 0.05) and 80% power. The threshold of sample size for 80% power is *n* = 32, *n* = 20, and *n* = 6 for minimum differences of 0.4 mm, 0.5 mm, and 1 mm, correspondingly [18]. The sample size in our study was far beyond *n* = 32 and thus provided a power higher than 80%. Bland-Altman analysis was performed to qualify the differences (bias, limits of agreement) between different examiners. Box and whisker plots were used to compare ultrasound measurements and clinical measurements among the Stage III periodontitis group and the gingivitis and Stage I periodontitis group.

## Results

**Fig. 1:**
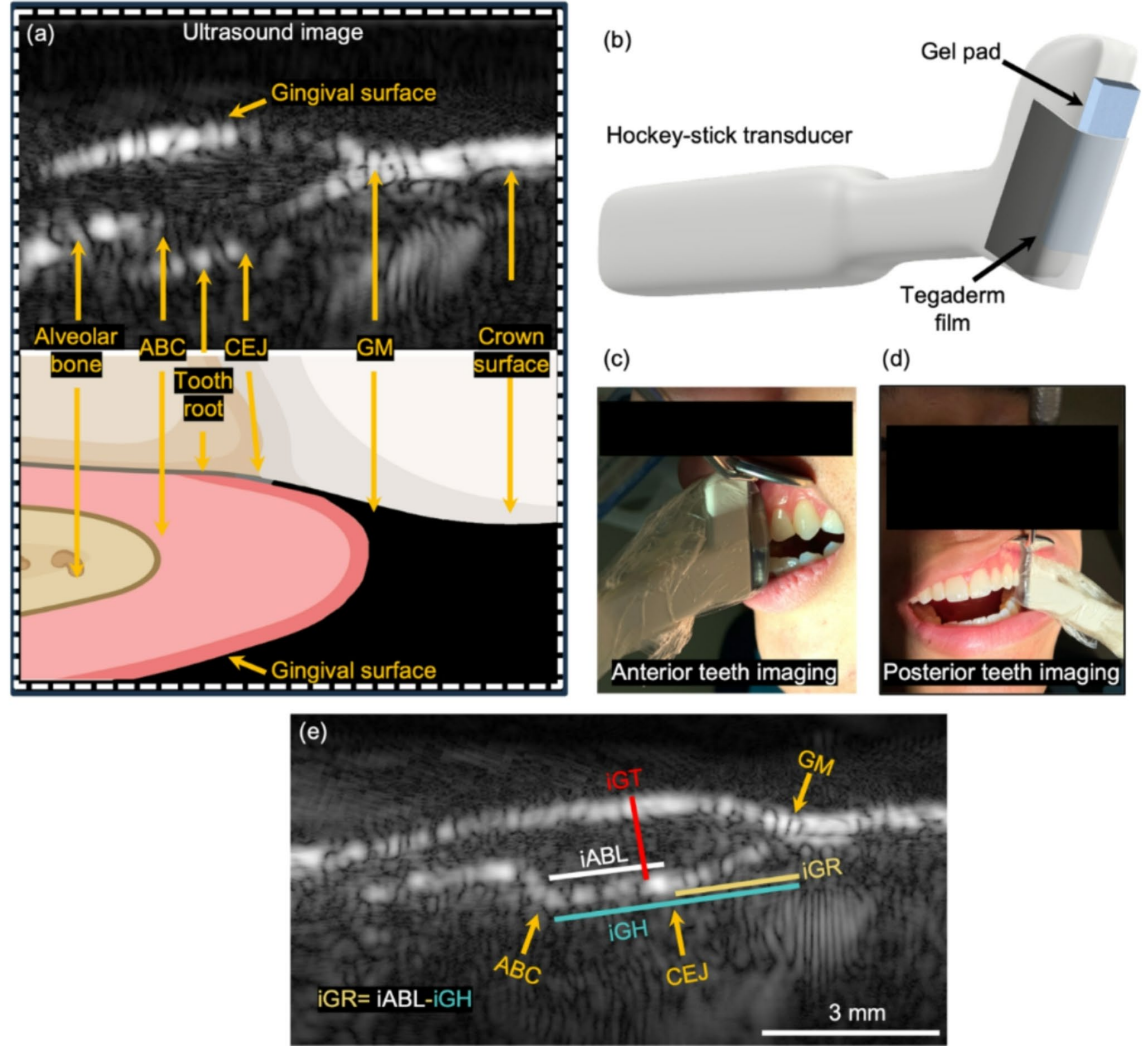
Periodontal ultrasound imaging via a hockey-stick transducer. (**a**) Illustration (top) and Ultrasound image (bottom) of the human tooth in a side-by-side view. Several tooth structures are resolvable in ultrasound imaging including the gingival margin (GM), alveolar bone crest (ABC), cementoenamel junction (CEJ), alveolar bone, root surface, and tooth surface. (**b**) Setup of a CL15-7 hockey-stick transducer for periodontal imaging. The gel pad offsets the tissue from the transducer for ultrasound focusing and coupling purposes. The Tegaderm film makes contact with the tissue. (**c**) (**d**) Illustrations of anterior teeth imaging and posterior teeth imaging, respectively. The angled design and the small size of the hockey-stick transducer allows it to image posterior teeth. No ultrasound gel was used on the tissue surface. (**e**) B-mode ultrasound image of tooth showing the definitions of periodontal characteristics including iGH, iABL, iGR, and iGT. The value of iGR becomes positive when CEJ is exposed and negative when the gingiva is coronal to the CEJ

Figure 1a shows a representative ultrasound image of a tooth and its associated periodontium (top) and a graphic depiction of these structures (bottom). Figure 1b shows the 9-MHz hockey-stick transducer (CL15-7, Philips) used in this study. The hockey-stick transducer was designed for 1 cm deep superficial imaging, such as human skin, with approximately 170-µm resolution. The ABC, CEJ, and GM are usually within 5 mm of the gingival surface; they are located at an ideal imaging depth of the transducer.

The angled design of the hockey-stick transducer allows its access to posterior teeth (Fig. 1c and d). To re-purpose the hockey-stick transducer for periodontal imaging, a 4-mm thick gel pad was placed underneath the transducer wrapped by a Tegaderm film. The gel pad offsets the tissue into the focus area of the hockey-stick transducer and facilitates ultrasound coupling. In general, ultrasound gel is needed for ultrasound coupling in clinical ultrasound imaging. However, the gel pad with Tegaderm (single use per subject) is pressed and moulded around the gingiva and tooth surface to provide good coupling without ultrasound gel, significantly improving the clinical experience.

Four imaging-based characteristics were defined based on the landmarks (Fig. 1e). (1) The distance from GM to CEJ is the imaged-based gingival recession (iGR), which can be either negative (i.e., the gingival margin is above the CEJ without recession) or positive (i.e., the gingival margin is below the CEJ and clinically defined as recession) in values. (2) The distance from ABC to CEJ is defined as image-based alveolar bone loss (iABL) for assessing bone loss. (3) The distance from ABC to GM is defined as image-based gingival height (iGH). (4) iGT is the thickness of gingival tissue measured at the level of the CEJ.

**Fig. 2:**
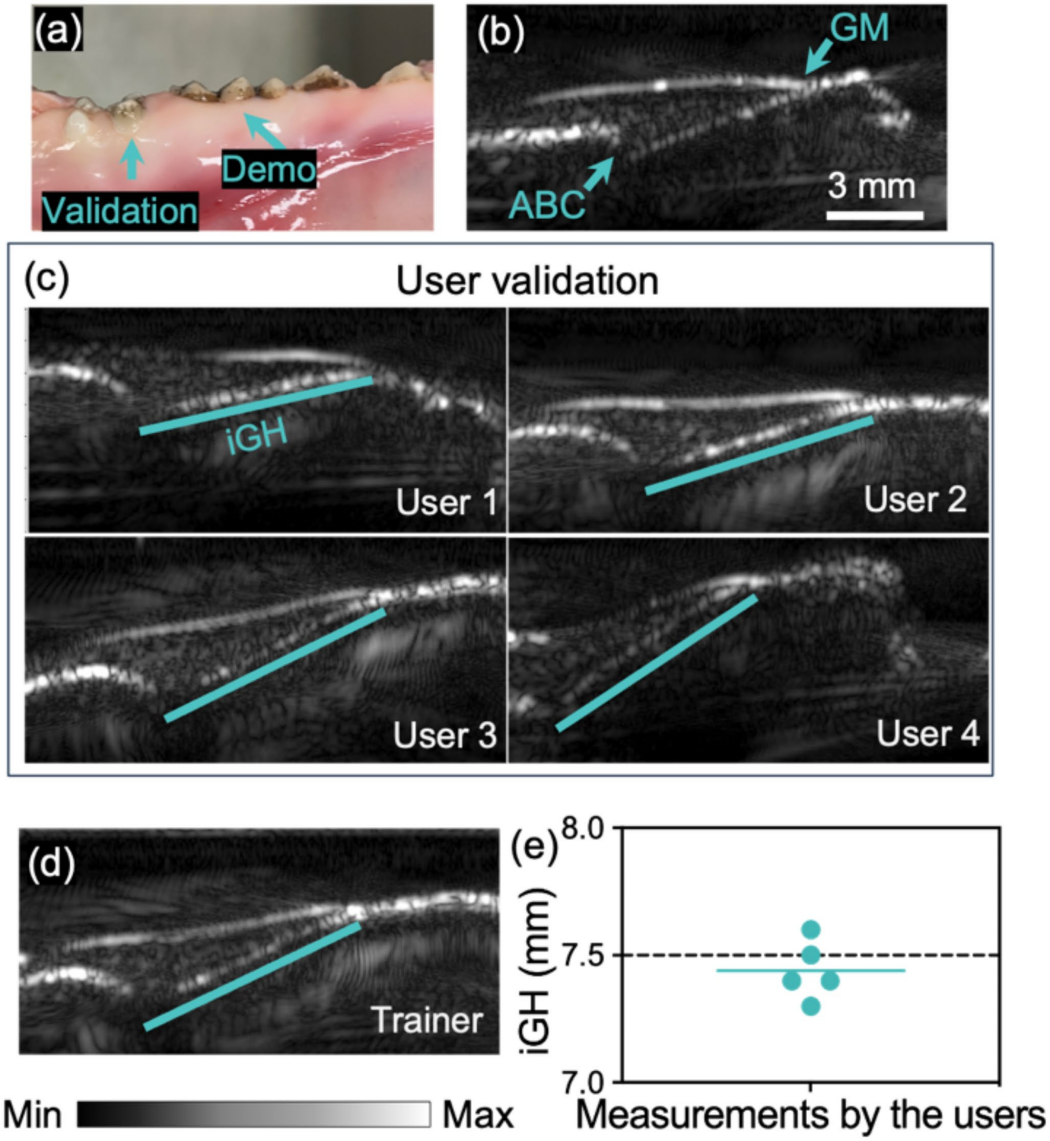
Ex-vivo inter-examiner test of ultrasound examiners. (**a**) A swine jaw image with teeth was used in the inter-examiner test. (**b**) Ultrasound image of a demo tooth. This demo image is obtained by an ultrasound researcher for the training of five inexperienced dental students. CEJ is not visible in the image. (**c**) Validation images obtained by the dental students. The students scanned the validation tooth blind to each other. (**d**) The trainer scanned the validation tooth as standard. (**e**) iGH values measured from the validation tooth by the students. The dashed line represents the iGH value measured from the trainer image

### Inter-rater reliability

To ensure all the clinical measurements were reliable even for new learners, we evaluated the inter-examiner variations with ex vivo swine teeth (Fig. 2). Five dental students without prior experience in ultrasound imaging were recruited. The ultrasound researcher trained the students in the protocol and measured the iGH of a demo tooth (Fig. 2a and b). The students and the researcher then scanned a validation tooth blinded to each other (Fig. 2c and d). The iGH values obtained by the students and the trainer are shown in Fig. 2e. All iGH values from the learners were close to the measurement by the ultrasound researcher, with a standard deviation (SD) of less than 0.2 mm, which is close to prior report [25]. Note that the CEJ could not be identified from the ultrasound images. For this reason, iABL and iGR were not measured. Nevertheless, these results demonstrated that the protocol for scanning was easy to learn and the variation of the imaging process is relatively small.

### Comparing ultrasound imaging and periodontal probing

**Fig. 3:**
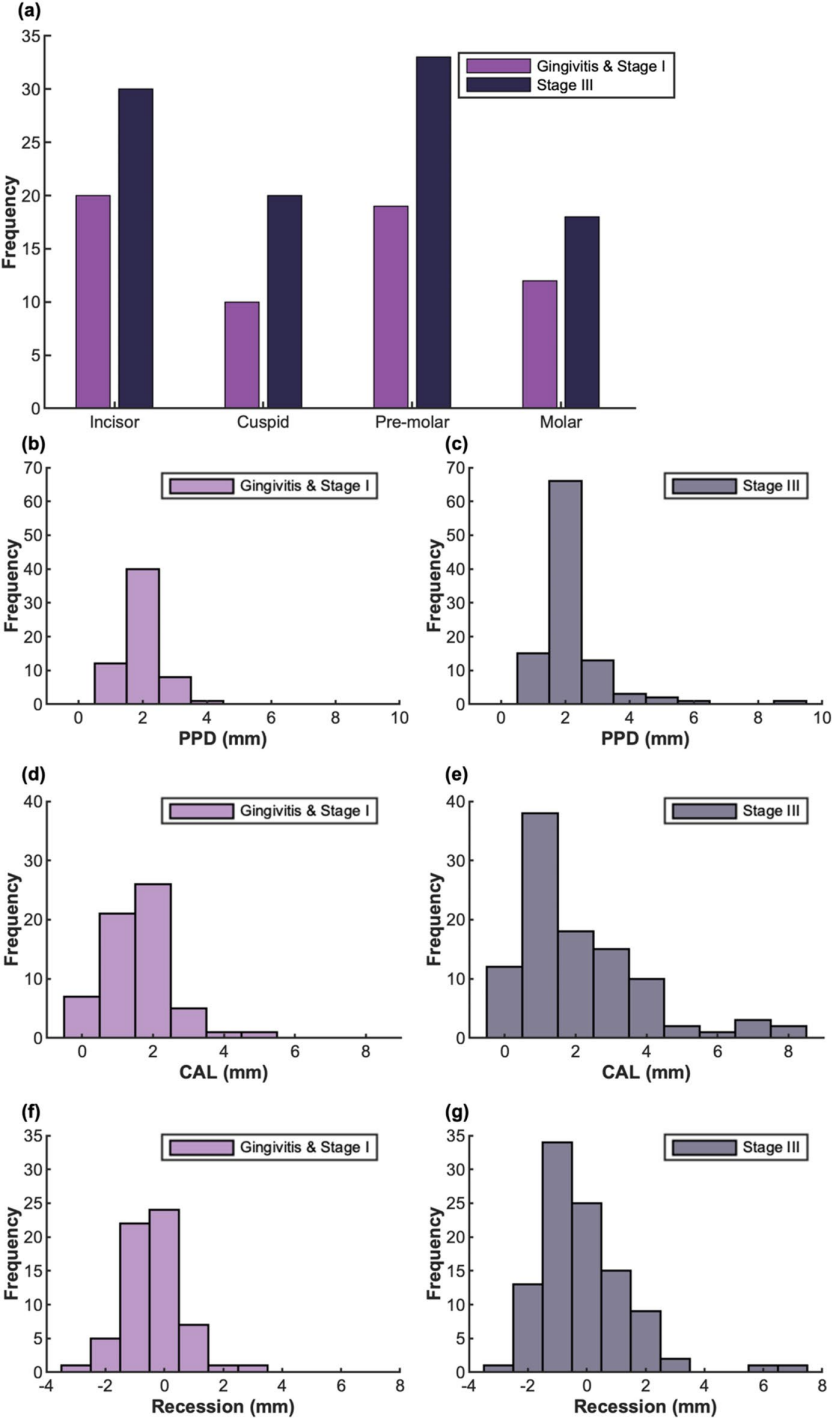
Distribution of periodontal measurements. (**a**) The number of teeth in each tooth type included in the study. Purple bars represent the distribution of the Gingivitis and Stage I group, and gray bars represent the distribution of the Stage III group. (**b**) and (**c**) are the distribution of PPD measurements, (**d**) and (**e**) are the distribution of CAL measurements, (**f**) and (**g**) are the distribution of gingival recession measurements

**Fig. 4:**
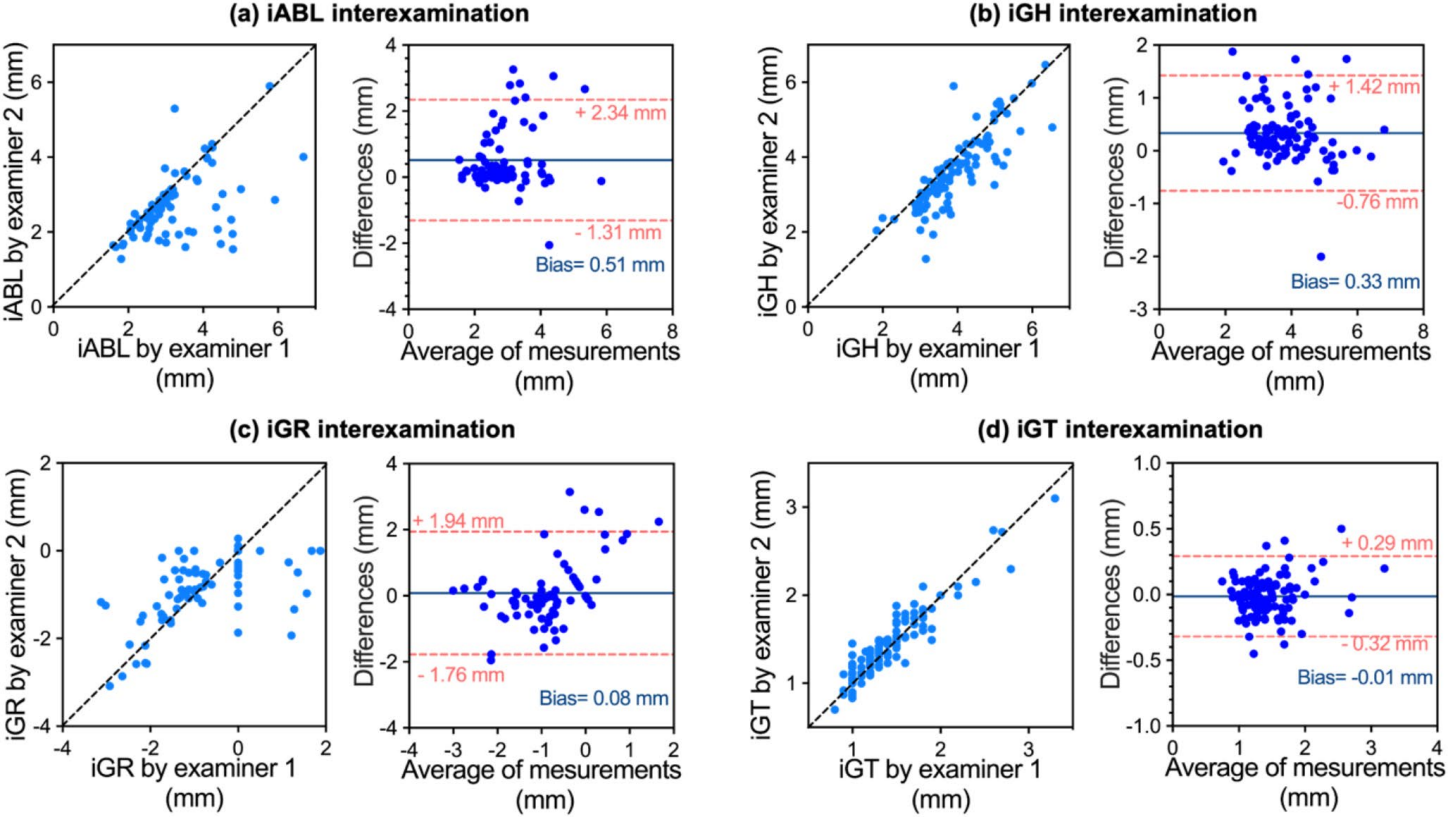
Inter-examiner variability. The ultrasound image-based measurements (iABL, iGH, iGR, and iGT) were compared between two examiners. (**a**) (**b**) (**c**) (**d**) The left panels are comparisons of iABL, iGH, iGR, and iGT measurements by the two examiners, respectively. The x-axis represents the measurements by Examiner 1, and the y-axis represents the measurements by Examiner 2. The right panels are the Bland-Altman analysis of the statistics in the left panels, correspondingly. The two dashed lines are the 95% limit of agreement

**Fig. 5:**
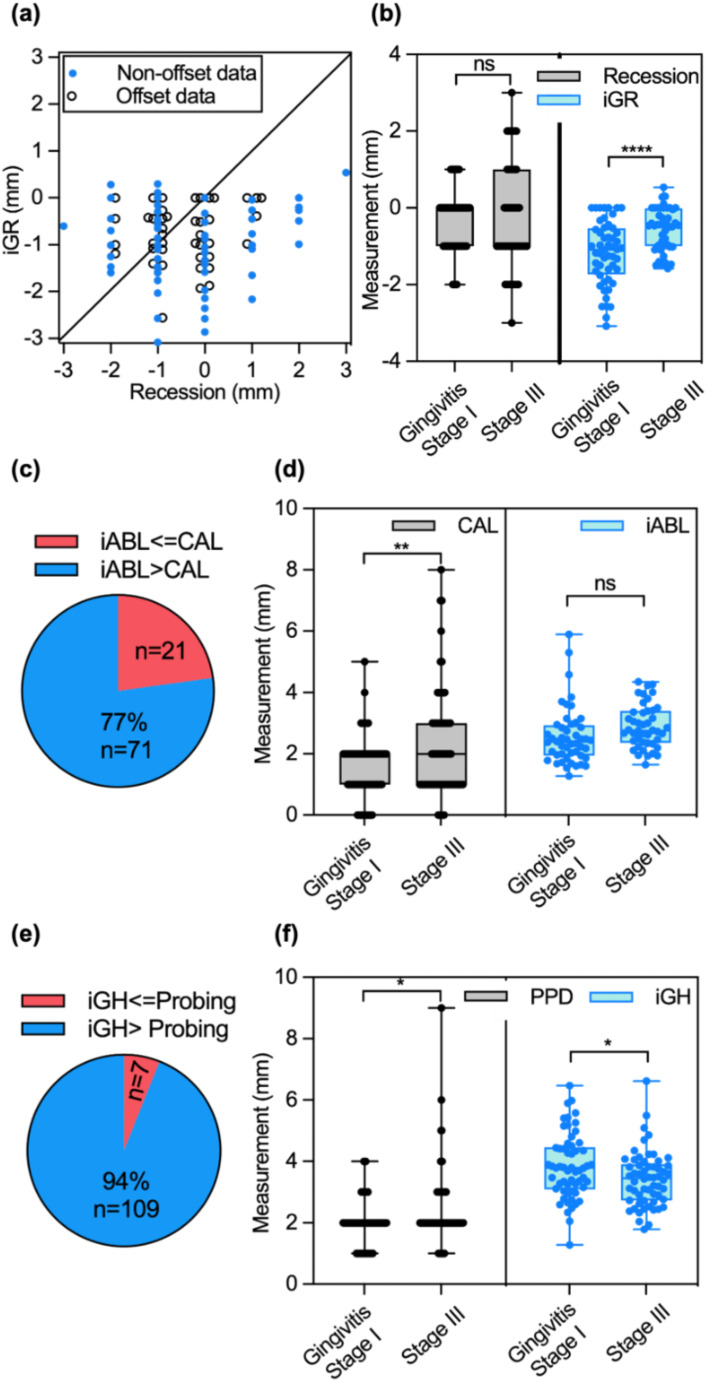
Comparison between ultrasound image-based measurements and clinical probing-based measurements. (**a**) Comparison between iGR measurements and gingival recession measurements tooth-by-tooth of all the subjects. The x-axis represents the gingival recession measurements by clinical probing, and the y-axis represents the iGR measurements by ultrasound; positive values indicate gingival recession. Note that we offset the position of some data points laterally to avoid data points from overlaying to each other for display purposes only. The data set includes 46 pre-molars and molars, and 54 incisors and canines. (**b**) Box-and-whisker plots of iGR and gingival recession in the gingivitis and Stage I periodontitis group and the Stage III periodontitis group. (**c**) The comparison between iABL and CAL tooth-by-tooth. iABL is expected to be larger than CAL. 77% of the iABL measurements are higher than the CAL values. (**d**) Box-and-whisker plots of iABL and CAL of the two groups. Stage III group has overall higher CAL and iABL values by 0.6 mm and 0.3 mm, respectively than the gingivitis and Stage I group. (**e**) The comparison between iGH and PPD. 94% of iGH values are higher than the corresponding PPD values. (**f**) Box-and-whisker plots of iGH and PPD of the two groups. Stage III group has overall higher probing depth by 0.4 mm and lower iGH values by 0.4 mm than the gingivitis and Stage I group. imaging may suggest better performance of this technique over conventional periodontal probing

A clinical study was performed to further evaluate the performance of the hockey-stick transducer. Thirteen human subjects were recruited. Seven of them were diagnosed with Stage III periodontitis, and six subjects were diagnosed with Stage I periodontitis and gingivitis. Each subject received periodontal examination by a periodontist (see Method 2.3). In total, 162 teeth were scanned including 53 pre-molars, 30 molars, and 79 incisors and canines. Two examiners independently measured iABL, iGH, iGR, and iGT from the same ultrasound images. First, a distribution of periodontal probing measurements is shown in Fig. 3. Second, the inter-examiner reliability of the measurements was determined (Fig. 4). Then, the measurements from ultrasound imaging were compared with those from the periodontal examination (Fig. 5).

### Distribution of probing measurements

A total number of 162 teeth were imaged, including 61 teeth from gingivitis and Stage I patients (12 molars, 19 premolars, 10 canines, and 20 incisors), and 101 teeth from Stage III patients (18 molars, 33 premolars, 20 canines, and 30 incisors) (Fig. 3a). PPD, CAL, and Gingival Recession were examined at six sites per tooth (see Methods). The hockey-stick transducer could only access to the mid-labial side. The lingual side was not accessible due to the dimensions and shape of the hockey-stick transducer used in this study. The mesial and distal sites were not imageable by ultrasound due to physical limitations–backscattered ultrasound from these regions is deflected away from the imaging plane. For this reason, clinical probing on the mid-labial side was used for comparison with ultrasound imaging. Figure 3b and g show the three distributions of the probing measurements, categorized into PPD (Fig. 3b and c), CAL (Fig. 3d and e), and Gingival Recession (Fig. 3f and g). The left panels correspond to gingivitis and Stage I periodontitis, and the right panels represent the Stage III group. All the measurements show a broader distribution above 5 mm in Stage III group than gingivitis and stage I group, indicating more severe disease.

### Inter-examiner reliability

Two blinded examiners with experience in ultrasound periodontal imaging independently identified the landmarks on the images and measured iABL (Fig. 4a), iGH (Fig. 4b), iGR (Fig. 4c), and iGT (Fig. 4d). The measurements by the examiners were compared tooth-by-tooth (left panels). The measurements were correlated with data points close to the line of equality (y = x). No significant differences were found between examiners for iGR and iGT (paired Student’s t-test, two-tailed; *p* > 0.05). However, we noted statistically differences for iABL and iGH measurements between examiners (paired, two-tailed t-test, *p* < 0.0001 for iABL, and *p* < 0.0001 for iGH). Nevertheless, the standard deviations (SD) of the differences between the two examiners were 0.93 mm for iABL, 0.56 mm for iGH, 0.95 mm for iGR, and 0.16 mm (iGT), which are smaller than the SDs of clinical inter-examiner tests [7, 26]. The right panels in Fig. 4 are the corresponding Bland-Altman analysis, thus further validating the agreement between the two examiners. The biases between the two examiners were 0.51 mm for iABL, 0.33 mm for iGH, 0.08 mm for iGR, and − 0.0146 mm for iGT. The greater biases between examiners for iABL and iGH might because the iABL and iGH values are inherently much larger than the iGR and iGT values (Fig. 1e).

The iGR, iABL, and iGH measurements were compared with the gingival recession, CAL, and probing depth measurements, respectively (Fig. 5). Figure 5a shows the gingival recession measurements of all the teeth obtained by probing (x-axis) and ultrasound imaging (y-axis). Bland-Altman analysis shows that the bias between iGR values and gingival recession are 0.54 mm, and the SD difference is 1.2 mm. Student’s t-test shows a statistically significant difference between the two groups of measurements (paired, two-tailed t-test, *p* = 0.0142). Note that we offset the position of some data points laterally to avoid data points from overlaying each other for display purposes only. The data points are distributed on the integer ticks along the x-axis because the read-outs of periodontal probing are integers with lower precision than ultrasound imaging (y-axis), i.e., probing is a categorical variable and imaging metric is a continuous variable.

Interestingly, we noticed that periodontal probing identified more cases of gingival recession than ultrasound imaging. The differences may be attributed to different interpretations of the location of CEJ by probing and imaging. Clinically visible CEJ may not be identical to the CEJ identified by imaging. Further studies are needed to test this hypothesis.

Analyses of the gingival recession were performed in two groups of subjects: the gingivitis and Stage I periodontitis group and the Stage III periodontitis group (Fig. 5b). In periodontal probing, the average gingival recession measurements were − 0.36 mm for the gingivitis and Stage I periodontitis group, and − 0.31 mm for the Stage III periodontitis group; the differences were not statistically significantly different (Student’s t-test, unpaired, two-tailed, *p* = 0.82). In contrast, the average iGR measurements were − 1.12 mm for the gingivitis and Stage I periodontitis group and − 0.56 mm for the Stage III periodontitis group; ultrasound imaging shows the differences were statistically significantly different (Student’s t-test, unpaired, two-tailed, *p* < 0.0001). The gingival recession measurement (positive or negative) is the distance between CEJ and the gingival margin. The measurements are expected to be greater in severe periodontitis than in gingivitis or mild periodontitis. The detection of greater measurements of gingival recession by ultrasound.

The iABL and iGH measurements by ultrasound imaging were compared with clinical CAL and PPD measurements, respectively. CEJ is a landmark of both iABL and CAL. The only difference is that iABL terminates at the ABC while CAL terminates at the apex of the gingiva sulcus by probing. The iABL values are always greater than the CAL values as shown in Fig. 1e. The comparison of iABL and CAL values showed that 77% of iABL measurements were greater than the CAL measurements as expected (Fig. 5c). We then compared the iABL and CAL measurements between the two groups of subjects: gingivitis and Stage I periodontitis group and the Stage III group (Fig. 5d). Gingivitis and Stage I periodontitis group are expected to have less periodontal destruction than the stage III patients. We noted statistically significant differences in CAL of the gingivitis and Stage I periodontitis group (mean ± SD 1.5 ± 0.99 mm) and the Stage III patients (mean ± SD 2.1 ± 1.7 mm) (Student’s t-test, *P* < 0.05). The iABL for the gingivitis and Stage I periodontitis group (mean ± SD 2.6 ± 0.94 mm) was less than the Stage III patients (mean ± SD 2.9 ± 0.7 mm). Still, the differences were not statistically significant (unpaired, two-tailed t-test, *p* = 0.0598).

The iGH measurements and PPDs are not identical, but the iGH should be a larger value assuming the periodontal pocket would not go beyond ABC. Indeed, the results showed that 94% of the iGH values were greater than PPDs (Fig. 5e). A few cases where iGH measurements were lower than PPDs were likely due to the rounding up of the PPDs. We further compared the iGH and PPD measurements between the gingivitis and Stage I periodontitis group, and the Stage III periodontitis group (Fig. 5f). The average PPDs were 1.9 mm (SD = 0.7 mm) for the gingivitis and Stage I periodontitis group, and 2.3 mm (SD = 1.1 mm) for the Stage III periodontitis group. The deeper PPDs in patients with severe periodontitis were as expected. However, similar trends were not observed for iGH measurements. The iGH measurements were 3.8 ± 1.1 mm for the gingivitis and Stage I periodontitis group, and 3.4 ± 0.9 mm for the Stage III periodontitis group. Student’s t-test shows that both PPD and iGH measurements were significantly different (*P* < 0.05) between the gingivitis and Stage I periodontitis group, and the Stage III periodontitis group (unpaired, two-tailed t-test, *p* = 0.0179 for PPD, and *p* = 0.0249 for iGH). Since iGH measures the distance between GM and ABC, both landmarks could recede during the progression of periodontitis. The results suggested that in severe periodontitis, GM might recede faster than ABC compared to mild periodontitis.

### Assessing risk for gingival recession

**Fig. 6:**
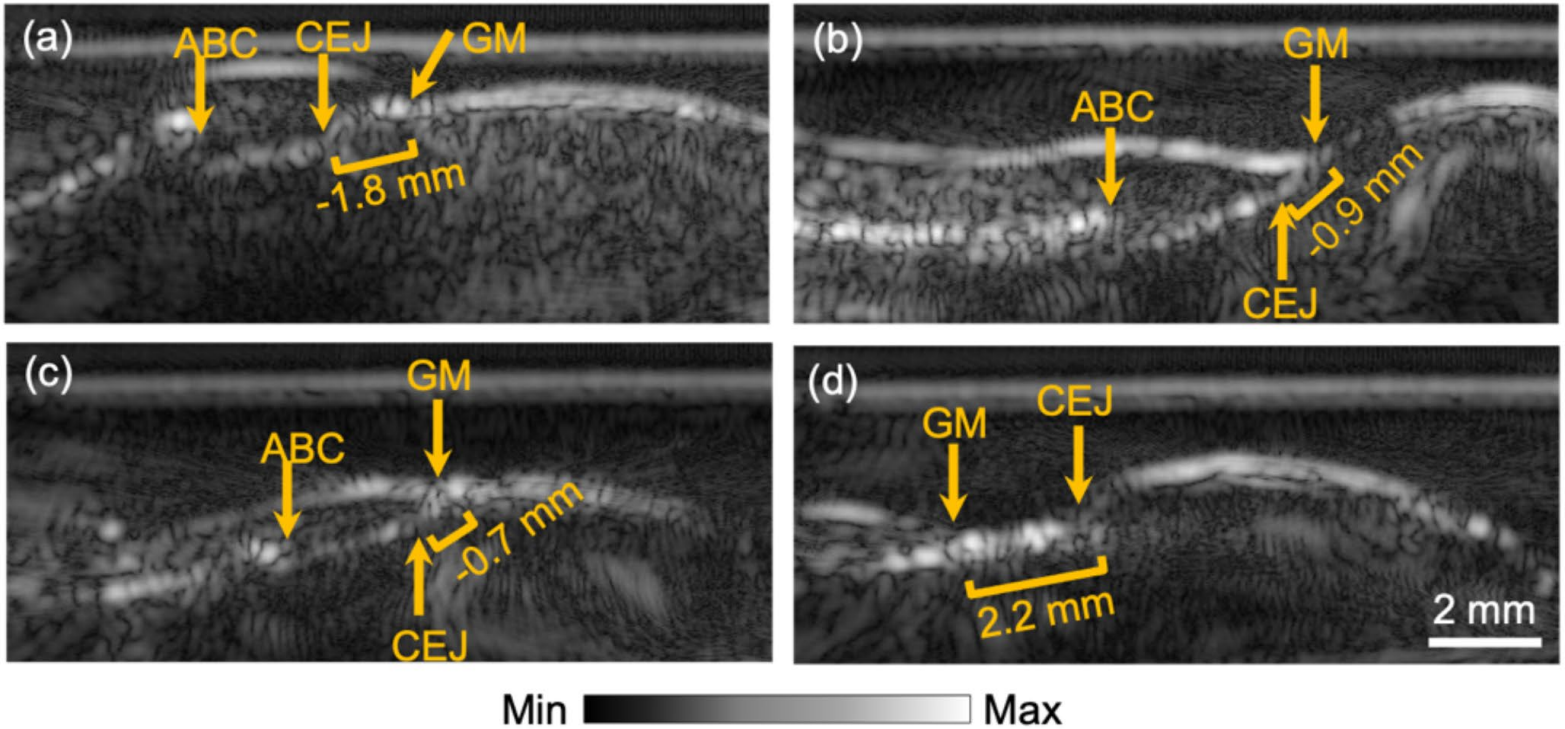
Four typical tooth images showing different levels of gingival recession. (**a**) An ultrasound image of incisor #8. (**b**) 1st molar #30. (**c**) 1st premolar #12. (**d**) Canine #6. The imaging-based gingival recession (iGR) were − 1.8 mm, -0.9 mm, -0.7 mm, and 2.2 mm for (**a**), (**b**), (**c**), and (**d**), respectively. Note that the value of iGR is positive in clinical recession, as shown in (**d**)

Clinically, the gingival recession is noted after the fact, i.e., it has occurred with sufficient tissue loss to expose the CEJ. Ultrasound imaging may provide a preview of the recession before it occurs. Figure 6 shows ultrasound images of teeth with different iGR values (from − 1.8 mm to 2.2 mm), including three cases without recession (Fig. 6a-c) and a case of clinical recession (Fig. 6d). The images reveal the relationships among GM, CEJ, and ABC, as well as the thickness and architecture of the marginal gingival. The clinicians may use ultrasound imaging to assess the recession risk or monitor marginal gingiva changes.

### Assessing reduction of gingival thickness

We note that the gingival thickness varies tooth by tooth, and between subjects suggesting that gingival thickness (iGT in ultrasound measurements) may also be influenced by other factors such as genetics. To explore this further, we quantified the correlations between iGT and iABL, iGT and iGH, and iGT and PPD from number of 98 teeth. Teeth with gingival recession were excluded from the analysis. The correlation coefficients are 0.17 between iGT and iABL, 0.33 between iGT and iGH, and 0.37 between iGT and PPD. The results suggest a weak correlation (0.1–0.39) between iGT and the other biomarkers (iABL, iGH, and PPD). We also anticipate that an overall thickness measurement across the area—rather than single-point assessments—could provide a more meaningful correlation with the clinical parameters, especially in tracking the development of periodontitis of individual subjects.

## Discussion

In this work, a 9-MHz hockey-stick transducer was used because of its angle design and relatively small footprint, which allow us to image molars and pre-molars. However, the resolution of a 9-MHz transducer is quite poor (~ 170 μm) [21]. In general, a transducer with a higher central frequency (> 30 MHz) can provide higher resolution but with the trade-off of decreased imaging depth. For example, a 30-MHz transducer can image less than 2-cm deep in tissue with around 50-µm resolution [27, 28]. However, periodontal biomarkers are usually within 5 mm of the gingiva surface (Fig. 1e), and thus high-frequency transducers are better suited for periodontal imaging. Future work will involve the development of high-frequency and miniaturized transducers customized for periodontal applications.

Ultrasound gel was not used to couple ultrasound between gingival tissue and transducer in our study, which improved patient and operator experience because ultrasound gel tastes bad and is inconvenient. To facilitate ultrasound coupling without ultrasound gel, we used a 4-mm-thick gel pad (Fig. 1b) [23]. The gel pad is deformable under pressure and is covered with a sterile adhesive film (Tegaderm).

In this work, we proved the capability of ultrasound imaging in measuring gingival recession measurement (Fig. 5a and b; positive values in Fig. 5a indicate gingival recession). However, we also noticed that periodontal probing identified 18 more cases of gingival recession than ultrasound imaging (out of 100 total cases); (Fig. 5a). The limited performance of imaging may be due in part to challenges in identifying the CEJ in ultrasound images. Indeed, the CEJ is a critical landmark for measuring gingival recession. This work used an ultra-low-cost and low-frequency transducer. In turn, the spatial resolution and image quality was moderate. This made identification of the CEJ difficult, which in turn could limit the ability to identify anatomical landmarks and calculate imaging biomarkers such as iGR. Similarly, we did not see a difference in the iABL values of early and advanced periodontitis (Fig. 5d), which is in contrast to our prior work [18] with a larger and higher-frequency transducer where diseased subjects had higher iABL values. The differences may be attributed to the variation of periodontal destruction of individual teeth in patients with similar periodontal diagnoses.

Computer vision and machine learning are emerging hotspot in medical imaging interpretation [31]. In this work, only 2D imaging and 1D analysis– i.e., measuring distances between different landmarks were performed, which does not fully leverage the 3D capabilities of ultrasound imaging for visualizing landmarks and periodontal characteristics. Indeed, 3D imaging would impose greater challenges in imaging interpretation. Manual interpretation of ultrasound images can be time-consuming and subjective. Like other medical imaging techniques, there is a great potential of integrating computer vision and machine learning algorithms to assist in interpreting gingival landmarks and periodontal characteristics, thereby improving diagnostic accuracy and reproducibility [32, 33].

Moreover, functional ultrasound may offer deeper insight into the diagnosis of periodontal disease [34]. Periodontal disease and recovery are associated with gingival inflammation. In this work, only structural information from ultrasound was used to identify periodontal landmarks. In comparison, functional ultrasound which could capture information about hemodynamics and tissue perfusion, remains a promising technique in dental applications [35]. Future work could explore functional ultrasound for assessing gingival inflammation and vascular responses.

Besides periodontal imaging, ultrasound imaging offers a way to assess the health of dental implants [36, 37]. Although X-rays provide good visualization of bone and implants, it lacks the contrast to assess soft tissue. Doppler ultrasound could detect early vascular changes, providing insights into the healing process at implant sites and offering a promising method for real-time post-surgical monitoring. However, the same limitations in transducer design and ultrasound physics make it challenging to image all surfaces of the implant as in periodontal imaging.

Future work will utilize more compact and high-frequency imaging devices to directly compare the capability of periodontal probing and ultrasound imaging in CEJ identification. We also expect the application of machine learning in periodontal ultrasound imaging will further facilitate the identification of the CEJ as well as other landmarks [32]. We recently showed that machine learning tools can automatically interpret the images and report the location of anatomical landmarks and image-based biomarkers like the iGR, iABL, and iGH [33]. Such automation will streamline chairside use.

## Conclusion

In conclusion, we investigated using a hockey-stick transducer for periodontal assessment. The angled shape of the hockey-stick transducer facilitates imaging of posterior teeth. We compared the characteristics obtained from ultrasound imaging and probing-based clinical parameters. The results suggest that the hockey-stick transducer is effective in periodontal assessment and could be a tool for periodontal diagnosis. Future work will focus on developing high-frequency transducers with dimensions similar to the hockey-stick design for full-mouth imaging with high resolution.

## Electronic supplementary material

Below is the link to the electronic supplementary material.

Supplementary Material 1

## Data Availability

The data in this article are available upon request to the corresponding author.
