# Supplementary material for: In vivo periodontal ultrasound imaging via a hockey-stick transducer and comparison to periodontal probing: a proof-of-concept study
Source: Clin Oral Investig. 2025 Apr 26;29(5):275. doi: 10.1007/s00784-025-06346-w (PMC12033097; doi:10.1007/s00784-025-06346-w)
Supplement: Supplementary file 1 — Supplementary Material 1 [file 784_2025_6346_MOESM1_ESM.docx]

**Supplementary information**

***In vivo* Periodontal ultrasound imaging via a hockey-stick transducer and comparison to periodontal probing: A proof-of-concept study**

Lei Fu^1^, Jason J. Chang^2^, Khalid Alhezaimi^2^, Lekshimi Sasi^1^, Suhel Khan^1^, Baiyan Qi^3^, Casey Chen^2^, and Jesse V Jokerst^1,3,4,*^

^1^ Aiiso Yufeng Li Family Department of Chemical and Nano Engineering, University of California, San Diego, La Jolla, CA 92093, USA

^2^Herman Ostrow School of Dentistry, University of Southern California, 925 West 34th Street, Los Angeles, CA, USA

^3^Material Science and Engineering Program, University of California, San Diego, La Jolla, CA 92093, USA

^4^Radiology Department, University of California, San Diego, La Jolla, CA 92093, USA

**
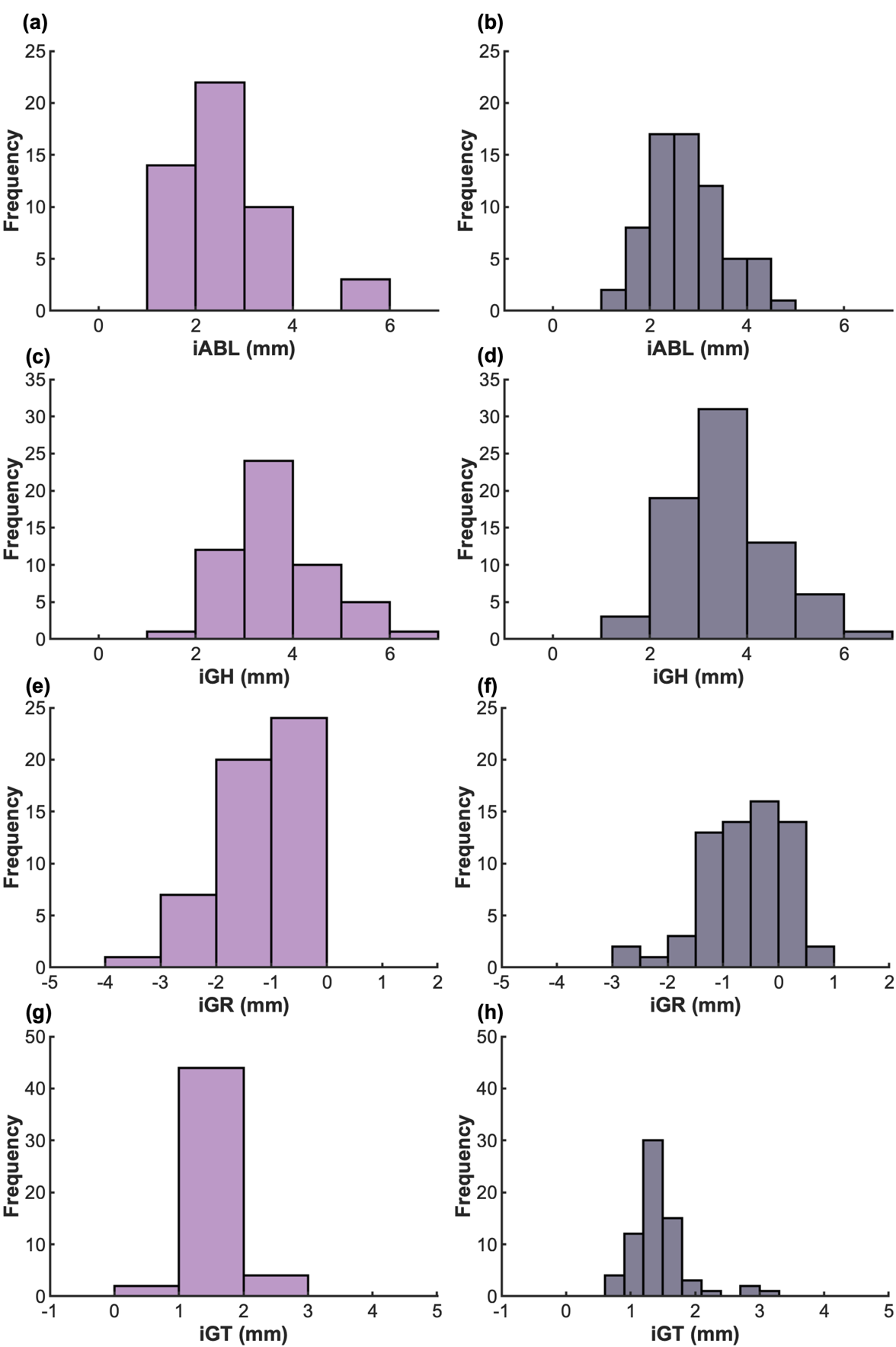
**

**Fig. 1 Distribution of imaging-based periodontal measurements.** (a) and (b) are the distribution of iABL measurements, (c) and (d) are the distribution of iGH measurements, (e) and (f) are the distribution of iGR measurements. (g) and (h) are the distribution of iGT measurements. Purple bars represent the distribution of the Gingivitis and stage I group and gray bars represent that of the Stage III group.

**Table of Contents**

**Summary of ultrasound and probing measurements**

|  |  |  | Ultrasound measurement (mm) | | | | Clinical measurement (mm) at mid-labial site | | | |
| --- | --- | --- | --- | --- | --- | --- | --- | --- | --- | --- |
| Patient Number | Index | Tooth Number | iABL | iGH | iGR | iGT | PPD | CAL | Recession | Bleeding on probing |
| 1  (Stage III) | 1 | 3 | - | - | - | - | 2 | 3 | 1 | - |
|  | 2 | 4 | 2.67 | 2.45 | 0.3 | 1.34 | 2 | 1 | -1 | - |
|  | 3 | 6 | 3.4 | 3.4 | 0 | 1.34 | 2 | 1 | -1 | - |
|  | 4 | 7 | - | 3.67 | - | - | 2 | 3 | 1 | - |
|  | 5 | 9 | 3.8 | 4.86 | -1.01 | 1.74 | 3 | 1 | -2 | - |
|  | 6 | 10 | - | 4.82 | - | - | 2 | 1 | -1 | - |
|  | 7 | 11 | 3.16 | 3.83 | -0.86 | 1.44 | 2 | 1 | -1 | - |
|  | 8 | 12 | 2.68 | 3.18 | 0 | 1.35 | 2 | 1 | -1 | - |
|  | 9 | 13 | 4 | - | - | - | 2 | 1 | -1 | - |
|  | 10 | 14 | - | - | - | - | 3 | 3 | 0 | - |
| 2  (Stage III) | 11 | 3 | - | - | - | - | 2 | 2 | 0 | - |
|  | 12 | 4 | - | - | - | - | 2 | 1 | -1 | - |
|  | 13 | 5 | 3.8 | 3.2 | 0.6 | 2.3 | 2 | 2 | 0 | - |
|  | 14 | 6 | 2.2 | 3.52 | -1.4 | 1.5 | 2 | 1 | -1 | - |
|  | 15 | 8 | 3 | 3.5 | -0.5 | 1.74 | 2 | 2 | 0 | - |
|  | 16 | 11 | 3.5 | 4.05 | -0.6 | 1.39 | 4 | 1 | -3 | - |
|  | 17 | 12 | 3 | 3.82 | -1.01 | 1.85 | 2 | 1 | -1 | - |
|  | 18 | 13 | - | - | - | - | 2 | 1 | -1 | - |
|  | 19 | 14 | 3.96 | 4.14 | -0.2 | 1.82 | 2 | 4 | 2 | - |
| 3  (Stage III) | 20 | 2 | - | - | - | - | 3 | 4 | 1 | - |
|  | 21 | 4 | - | - | - | - | 2 | 4 | 2 | - |
|  | 22 | 5 | 4.23 | 4.23 | 0 | 1.23 | 2 | 1 | -1 | - |
|  | 23 | 6 | - | - | - | - | 2 | 0 | -2 | - |
|  | 24 | 7 | 2.1 | 4.12 | -1.91 | 1.3 | 2 | 0 | -2 | - |
|  | 25 | 8 | - | 4.23 | - | 1.79 | 2 | 1 | -1 | - |
|  | 26 | 9 | 2.01 | 3.47 | -1.47 | 1.53 | 2 | 0 | -2 | - |
|  | 27 | 10 | 2.23 | 3.12 | -1 | 1.45 | 2 | 0 | -2 | - |
|  | 28 | 11 | 2.35 | 2.62 | -0.4 | 1.47 | 2 | 1 | -1 | - |
|  | 29 | 12 | 2.65 | 3.48 | -0.8 | 1.36 | 1 | 1 | 0 | - |
|  | 30 | 13 | 2.38 | 2.38 | 0 | 1.23 | 2 | 2 | 0 | - |
| 4  (Stage III) | 31 | 3 | - | - | - | - | 2 | 3 | 1 | - |
|  | 32 | 4 | - | - | - | - | 2 | 0 | -2 | - |
|  | 33 | 5 | - | - | - | - | 2 | 1 | -1 | - |
|  | 34 | 6 | 1.83 | 3.59 | -1.47 | 1.57 | 2 | 1 | -1 | - |
|  | 35 | 7 | 2.1 | 3.2 | -1.1 | 1.4 | 1 | 0 | -1 | - |
|  |  |  |  |  |  |  |  |  |  |  |
|  | 36 | 11 | 2.32 | 2.76 | -0.44 | 0.91 | 2 | 0 | -2 | - |
|  | 37 | 12 | 3.23 | 2.94 | -0.3 | 0.83 | 2 | 1 | -1 | - |
|  | 38 | 13 | 2.23 | 1.79 | -0.44 | 1.06 | 2 | 1 | -1 | - |
|  | 39 | 14 | - | - | - | - | 4 | 4 | 0 | - |
|  | 40 | 22 | - | - | - | - | 1 | 8 | 7 | - |
|  | 41 | 23 | - | - | - | - | 2 | 4 | 2 | - |
|  | 42 | 24 | 2.73 | 2.73 | 0 | 1.06 | 1 | 4 | 3 | - |
|  | 43 | 25 | 3.37 | 3.11 | -0.26 | 1.3 | 1 | 4 | 3 | - |
|  | 44 | 26 | - | - | - | - | 1 | 1 | 0 | - |
|  | 45 | 27 | - | - | - | - | 1 | 7 | 6 | - |
| 5  (Stage I) | 46 | 11 | 3.15 | 3.8 | -0.65 | 1.21 | 1 | 0 | -1 | - |
|  | 47 | 12 | - | - | - | - | 1 | 0 | -1 | - |
|  | 48 | 13 | 2.21 | 3.4 | -1.19 | 1.3 | 2 | 0 | -2 | + |
|  | 49 | 14 | 5.9 | 5.9 | 0 | 2.15 | 4 | 2 | -2 | - |
|  | 50 | 18 | 3.33 | 5.3 | -1.97 | 1.32 | 2 | 2 | 0 | + |
|  | 51 | 19 | - | - | - | - | 1 | 1 | 0 | + |
|  | 52 | 20 | 1.26 | 2.23 | -0.97 | 1 | 2 | 1 | -1 | + |
|  | 53 | 21 | 2.26 | 3.03 | -0.77 | 1.1 | 2 | 3 | 1 | - |
|  | 54 | 22 | - | - | -2.36 | 1.12 | 1 | 1 | 0 | - |
|  | 55 | 23 | 1.6 | 2.7 | -1.1 | 0.83 | 2 | 1 | -1 | - |
|  | 56 | 24 | 2.18 | 4.34 | -2.16 | 1.18 | 1 | 2 | 1 | - |
|  | 57 | 25 | - | - | -1.15 | - | 2 | 1 | -1 | - |
|  | 58 | 26 | 1.67 | 2.32 | -0.65 | 1.38 | 2 | 2 | 0 | - |
|  | 59 | 27 | - | - | - | - | 2 | 1 | -1 | - |
|  | 60 | 28 | 1.89 | 2.68 | -0.79 | 0.7 | 1 | 1 | 0 | - |
|  | 61 | 29 | 1.28 | 1.28 | 0 | 1.1 | 1 | 2 | 1 | - |
|  | 62 | 30 | - | - | - | - | 3 | 2 | -1 | - |
|  | 63 | 31 | - | 3.89 | - | - | 3 | 1 | -2 | - |
| 6  (Stage III) | 64 | 3 | - | - | - | - | 5 | 7 | 2 | - |
|  | 65 | 4 | - | - | - | - | 2 | 1 | -1 | - |
|  | 66 | 5 | 2.75 | - | - | - | 2 | 2 | 0 | - |
|  | 67 | 6 | 4.26 | 4.7 | -0.44 | 1.23 | 3 | 1 | -2 | - |
|  | 68 | 7 | 2.42 | 2.42 | 0 | 1.27 | 2 | 3 | 1 | - |
|  | 69 | 10 | 2.49 | 2.91 | -0.42 | 3.1 | 2 | 1 | -1 | - |
|  | 70 | 11 | - | - | - | - | 2 | 1 | -1 | - |
|  | 71 | 12 | 1 | 1.97 | -1 | 1.3 | 3 | 3 | 0 | - |
|  | 72 | 13 | 2.38 | 3.08 | -0.7 | 1.2 | 3 | 2 | -1 | - |
|  | 73 | 14 | - | - | - | - | 6 | 6 | 0 | - |
|  | 74 | 19 | - | 6.62 | - | 2.74 | 9 | 8 | -1 | + |
|  | 75 | 20 | 2.95 | 2.67 | +0.28 | 1.23 | 3 | 1 | -2 | + |
|  | 76 | 21 | 3.34 | 3.34 | 0 | 1.1 | 3 | 1 | -2 | + |
|  | 77 | 22 | 2.62 | 2.51 | +0.11 | 0.9 | 2 | 1 | -1 | - |
|  | 78 | 23 | 2.78 | 3.05 | -0.27 | 1.55 | 2 | 1 | -1 | - |
|  | 79 | 24 | - | - | - | - | 2 | 2 | 0 | - |
|  | 80 | 25 | - | - | - | - | 5 | 7 | 2 | + |
|  | 81 | 26 | - | - | - | - | 3 | 4 | 1 | - |
|  | 82 | 27 | 1.94 | 2.9 | -0.96 | 0.87 | 2 | 1 | -1 | - |
|  | 83 | 28 | - | - | - | - | 3 | 1 | -2 | - |
|  | 84 | 29 | - | 2.45 | - | - | 2 | 1 | -1 | - |
|  | 85 | 30 | 3.06 | 3.76 | -0.7 | 1.43 | 4 | 2 | -2 | - |
| 7  (Stage I) | 86 | 3 | - | - | - | - | 3 | 0 | -3 | - |
|  | 87 | 5 | 1.72 | 2.05 | -0.33 | 1.13 | 3 | 3 | 0 | - |
|  | 88 | 6 | 2 | 2.78 | -0.78 | 1.12 | 2 | 1 | -1 | - |
|  | 89 | 7 | 2.33 | 3.66 | -1.33 | 1.38 | 2 | 2 | 0 | - |
|  | 90 | 8 | 2.38 | 3.89 | -1.5 | 1.84 | 2 | 2 | 0 | - |
|  | 91 | 9 | 2.53 | 2.97 | -0.44 | 1.45 | 2 | 1 | -1 | - |
|  | 92 | 10 | 2.03 | 2.59 | -0.56 | 1 | 2 | 1 | -1 | - |
| 8  (Gingivitis) | 93 | 3 | 2.44 | 3.69 | -1.25 | 1.49 | 2 | 0 | -2 | - |
|  | 94 | 4 | 2.23 | 3.84 | -1.6 | 2 | 3 | 1 | -2 | - |
|  | 95 | 5 | 3.1 | 5.98 | -2.88 | 1.53 | 2 | 1 | -1 | - |
|  | 96 | 6 | 2.48 | 4.51 | -2.03 | 1.35 | 2 | 1 | -1 | - |
|  | 97 | 7 | 5.3 | 6.47 | -1.17 | 1.59 | 2 | 2 | 0 | - |
|  | 98 | 8 | - | 4.6 | - | - | 2 | 2 | 0 | - |
|  | 99 | 9 | 2.07 | 5.15 | -3.08 | 1.88 | 2 | 1 | -1 | - |
|  | 100 | 10 | - | 4.98 | 0 | 1.4 | 2 | 2 | 0 | - |
|  | 101 | 11 | 3.31 | 4.27 | -1 | 1.23 | 2 | 2 | 0 | - |
|  | 102 | 12 | 1.69 | 3.27 | -1.6 | 1.26 | 2 | 1 | -1 | - |
|  | 103 | 13 | 1.78 | 3.26 | -1.5 | 2.1 | 3 | 2 | -1 | - |
|  | 104 | 14 | 2.4 | 5.26 | -2.86 | 1.62 | 3 | 3 | 0 | - |
| 9  (Stage III) | 105 | 3 | 2.11 | 3.61 | -1.5 | 1.2 | 3 | 3 | 0 | - |
|  | 106 | 4 | 3.36 | 3.82 | -0.46 | 1.67 | 2 | 2 | 0 | - |
|  | 107 | 5 | 2.77 | 3.86 | -1.09 | 1.6 | 2 | 3 | 1 | - |
|  | 108 | 6 | 4.01 | 4.01 | 0 | 1.12 | 2 | 1 | -1 | - |
|  | 109 | 7 | 2.73 | 3.99 | -1.26 | 1.44 | 2 | 2 | 0 | - |
|  | 110 | 8 | 2.1 | 3.68 | -1.58 | 1.8 | 2 | 2 | 0 | - |
|  | 111 | 9 | 3.67 | 5.09 | -1.42 | 2.1 | 2 | 2 | 0 | - |
|  | 112 | 11 | - | 4.07 | - | - | 2 | 3 | 1 | - |
|  | 113 | 13 | 3.42 | 3.87 | -0.45 | 1.74 | 2 | 3 | 1 | - |
|  | 114 | 14 | 2.67 | 3.66 | -0.99 | 1.47 | 3 | 5 | 2 | - |
| 10  (Gingivitis) | 115 | 2 | 2.41 | 3.09 | -0.68 | 1.11 | 2 | 4 | 2 | - |
|  | 116 | 4 | 1.96 | 3.11 | -1.05 | 1.36 | 2 | 2 | 0 | - |
|  | 117 | 5 | 2.85 | 3.36 | -0.51 | 1.04 | 2 | 2 | 0 | - |
|  | 118 | 6 | 3.7 | 4.35 | -0.65 | 1.21 | 1 | 0 | -1 | - |
|  | 119 | 7 | 3.15 | 4.8 | -1.65 | 1.19 | 1 | 2 | 1 | - |
|  | 120 | 8 | 2.48 | 4.24 | -1.76 | 1.48 | 2 | 1 | -1 | - |
|  | 121 | 9 | 3.62 | 3.78 | -0.16 | 1.7 | 2 | 1 | -1 | - |
|  | 122 | 10 | 3.57 | 4.58 | -1.01 | 1.42 | 2 | 3 | 1 | - |
|  | 123 | 11 | 2.63 | 3.15 | -0.52 | 1.06 | 2 | 2 | 0 | - |
|  | 124 | 12 | 2.47 | 2.47 | 0 | 1.26 | 2 | 2 | 0 | - |
|  | 125 | 13 | 2.6 | 2.6 | 0 | 1.27 | 2 | 3 | 1 | - |
|  | 126 | 14 | 2.34 | 2.34 | 0 | 1.6 | 2 | 2 | 0 | - |
| 11  (Stage III) | 127 | 3 | 2.25 | 3.34 | -1.09 | 1.76 | 2 | 3 | 1 | - |
|  | 128 | 4 | 1.68 | 2.65 | -0.97 | 1.25 | 2 | 2 | 0 | - |
|  | 129 | 5 | - | - | - | - | 1 | 1 | 0 | - |
|  | 130 | 6 | 2.5 | 3.8 | -1.3 | 1.11 | 1 | 0 | -1 | - |
|  | 131 | 7 | - | 5.41 | - | 1.29 | 1 | 0 | -1 | - |
|  | 132 | 8 | 3.02 | 5.17 | -2.15 | 1.31 | 1 | 0 | -1 | - |
|  | 133 | 9 | 2.87 | 5.43 | -2.56 | 1.5 | 1 | 0 | -1 | - |
|  | 134 | 10 | 1.86 | 4.44 | -2.58 | 1.33 | 2 | 2 | 0 | - |
|  | 135 | 11 | 4.58 | 5.58 | -1 | 0.86 | 1 | 1 | 0 | - |
|  | 136 | 12 | 2.75 | 2.75 | 0 | 1.3 | 2 | 2 | 0 | - |
|  | 137 | 13 | 1.54 | 3.47 | -1.93 | 1.18 | 2 | 2 | 0 | - |
|  | 138 | 14 | 2.97 | 4.41 | -1.44 | 2.72 | 2 | 1 | -1 | - |
| 12 (Gingivitis) | 139 | 3 | 5.26 | 3.7 | -1.56 | 2 | 3 | 2 | -1 | - |
|  | 140 | 4 | 1.6 | 3.47 | -1.87 | 1.49 | 2 | 2 | 0 | - |
|  | 141 | 5 | 2.95 | 3.83 | -0.88 | 1.48 | 2 | 2 | 0 | - |
|  | 142 | 6 | 1.94 | 3.02 | -1.08 | 1.08 | 1 | 0 | -1 | - |
|  | 143 | 7 | - | - | - | - | 2 | 5 | 3 | - |
|  | 144 | 8 | 1.84 | 4.41 | -2.57 | 1.9 | 2 | 1 | -1 | - |
|  | 145 | 9 | 1.65 | 3.79 | -2.14 | 1.7 | 2 | 2 | 0 | - |
|  | 146 | 10 | 1.97 | 3.61 | -1.64 | 1.65 | 1 | 1 | 0 | - |
|  | 147 | 11 | 3.02 | 3.28 | -0.26 | 1.32 | 1 | 2 | 1 | - |
|  | 148 | 12 | 3.85 | 3.85 | 0 | 1.36 | 2 | 2 | 0 | - |
|  | 149 | 13 | 2.07 | 3.34 | -1.27 | 1.27 | 2 | 2 | 0 | - |
|  | 150 | 14 | - | 2.95 | - | - | 2 | 1 | -1 | - |
| 13  (Stage III) | 151 | 2 | - | - | - | - | 2 | 3 | 1 | - |
|  | 152 | 3 | 1.95 | 2.44 | -0.49 | 1.49 | 3 | 5 | 2 | - |
|  | 153 | 4 | 1.3 | 2.04 | -0.74 | 1.78 | 2 | 2 | 0 | - |
|  | 154 | 5 | 1.65 | 2.53 | -0.98 | 1.35 | 2 | 3 | 1 | - |
|  | 155 | 6 | 3.14 | 3.1 | -0.04 | 0.92 | 2 | 3 | 1 | - |
|  | 156 | 7 | - | 5.49 | - | - | 2 | 0 | -2 | - |
|  | 157 | 8 | 2.66 | 3.6 | -0.46 | 1.78 | 2 | 1 | -1 | - |
|  | 158 | 9 | 2.46 | 3.43 | -0.97 | 1.68 | 1 | 1 | 0 | - |
|  | 159 | 10 | 2.33 | 2.72 | -0.39 | 1.18 | 1 | 2 | 1 | - |
|  | 160 | 11 | 2.86 | 2.32 | +0.54 | 1.22 | 2 | 4 | 2 | - |
|  | 161 | 12 | 1.93 | 1.93 | 0 | 1.09 | 2 | 4 | 2 | - |
|  | 162 | 15 | 4.35 | 4.35 | 0 | 1.52 | 2 | 3 | 1 | - |
